# SETD2: an epigenetic modifier with tumor suppressor functionality

**DOI:** 10.18632/oncotarget.9368

**Published:** 2016-05-14

**Authors:** Jun Li, Gerben Duns, Helga Westers, Rolf Sijmons, Anke van den Berg, Klaas Kok

**Affiliations:** ^1^ Department of Genetics, University of Groningen, University Medical Center Groningen, The Netherlands; ^2^ Genome Sciences Centre, British Columbia Cancer Agency, Vancouver, Canada; ^3^ Department of Pathology and Medical Biology, University of Groningen, University Medical Center Groningen, The Netherlands

**Keywords:** SETD2, H3K36me3, ccRCC, histone modification, tumor suppressor gene

## Abstract

In the past decade important progress has been made in our understanding of the epigenetic regulatory machinery. It has become clear that genetic aberrations in multiple epigenetic modifier proteins are associated with various types of cancer. Moreover, targeting the epigenome has emerged as a novel tool to treat cancer patients. Recently, the first drugs have been reported that specifically target *SETD2*-negative tumors. In this review we discuss the studies on the associated protein, Set domain containing 2 (SETD2), a histone modifier for which mutations have only recently been associated with cancer development. Our review starts with the structural characteristics of SETD2 and extends to its corresponding function by combining studies on SETD2 function in yeast, Drosophila, *Caenorhabditis elegans*, mice, and humans. *SETD2* is now generally known as the single human gene responsible for trimethylation of lysine 36 of Histone H3 (H3K36). H3K36me3 readers that recruit protein complexes to carry out specific processes, including transcription elongation, RNA processing, and DNA repair, determine the impact of this histone modification. Finally, we describe the prevalence of *SETD2*-inactivating mutations in cancer, with the highest frequency in clear cell Renal Cell Cancer, and explore how SETD2-inactivation might contribute to tumor development.

## INTRODUCTION

In recent years, *SETD2* has attracted a lot of interest as a gene whose inactivation is involved in tumor initiation and progression. However, Faber *et al* [1] had already identified a protein encoded by *SETD2* in 1998 using a two-hybrid-based approach to search for proteins that interact with Huntingtin, the protein known to be associated with Huntington's disease (HD). They identified several candidates, three of which contained a WW domain. One of these three proteins was Huntingtin Yeast Partner B (HYPB). Around the same time Mao *et al* [2] and Zhang *et al* [3] identified and analyzed a large set of transcripts from human umbilical cord CD34+ hematopoietic stem/progenitor cells. One of these transcripts, *HSPC069*, had a sequence identical to *HYPB* and represented the same gene. A few years later, *HSPC069* was shown to contain an AWS-SET-PostSET domain and to possess histone methyl transferase activity specific for lysine 36 of histone 3 (H3K36) [4]. In a study focusing on proteins that interact with a DNA-binding motif in the E1A promoter, a transcript identical to *HYPB* was identified and named *HBP231* [5]. The associated gene is ubiquitously expressed in all tissues and cell lines tested, including many cancer-derived cell lines.

Edmunds *et al* [6] introduced the gene symbol *SETD2* in 2008, and made a more detailed analysis of the global and transcription-dependent distribution of tri-methylated histone H3 lysine 36 (H3K36me3) in mammalian cells. This was in line with the role of the *Saccharomyces cerevisiae* homologue of SETD2, ySET2, which had been identified in 2002 [7]. An important step in understanding the biology of ySET2 was its interaction with the serine2 phosphorylated C-terminal domain (CTD) of RNA polymerase II (RNA Pol II), linking ySET2 to the transcription elongation process [8]. A similar interaction was later confirmed for mammalian SETD2 [4, 9]. It was, however, not just its role in regulating transcription that attracted the interest of researchers over the years. The presence of inactivating mutations in a range of tumor types, most notably in clear cell renal cell cancer (ccRCC), sparked an additional focus of research: exploring the role of SETD2 in cancer development.

In this review the domains and functions of SETD2 in normal biology will be discussed in more detail. In the final part of the review, we focus on how loss of SETD2 function can contribute to cancer development.

## THE FUNCTIONAL DOMAINS OF SETD2

The human SETD2 gene is located at the cytogenetic band p21.31 of chromosome 3, a region frequently targeted by copy number loss in various tumors [10]. *SETD2* encompasses a genomic region of 147Kb, and the 21 exons encode an 8,452nt transcript. The SETD2 protein consists of 2,564 amino acids and has a molecular weight of 287.5 KD. Three conserved functional domains have been identified in the SETD2 protein: the triplicate AWS-SET-PostSET domains, a WW domain and a Set2 Rpb1 interacting (SRI) domain.

### AWS-SET-PostSET domain

The human SET domain is a motif of 130 amino acids that is evolutionarily conserved from mammals to yeast and even in some bacteria and viruses [11, 12]. The SET domain was identified by comparison of the protein sequence of the Drosophila position-effect variegation suppressor gene, Su(var)3-9, with the protein sequence of several other genes [13]. The acronym SET stands for “Suppressor of Variegation, Enhancer of zeste and Trithorax”, which are the three genes that led to the discovery of this domain.

The SET domain is usually present as part of a multi-domain, flanked by an AWS (Associated with SET) and a PostSET domain. Generally, SET-domain-containing proteins transfer one or several methyl groups from S-adenosyl-L-methionine to the amino group of a lysine or an arginine residue of histones or other proteins [14]. This transfer is dependent on the flanking AWS and PostSET regions, which contain several conserved cysteine residues. In contrast to other methyltransferases, SET-domain-containing methyltransferases have a α-sheet structure that facilitates multiple rounds of methylation without substrate disassociation [15].

### WW domain

The term “WW domain” was originally described in 1995 by Sudol *et al* [16] and refers to the presence of two conserved tryptophan (W) residues spaced 20-22 amino acids apart. Binding assays showed that the WW domain preferentially binds to proline-rich segments, mediating protein-protein interactions to participate in a variety of molecular processes [17]. The WW domain recognizes motifs like Proline-Proline-x-Tyrosine (PPxY) [18], phospho-Serine-Proline (p-SP) or phospho-Threonine-Proline (p-ST) [19], and mediates protein binding [20]. Aberrant expression of WW-domain-containing genes has been associated with different diseases such as HD [21], Alzheimer's disease [22], and multiple cancer subtypes [23, 24]. The WW domain in the C-terminal region of SETD2 interacts with the Huntingtin protein *via* its proline-rich segment, regardless of the length of the HD-associated polyglutamine track [1], and may also interact with TP53 [25]. Gao *et al* [26] performed a detailed nuclear magnetic resonance study on the interaction of SETD2 with Huntingtin. SETD2 contains a proline-rich stretch that precedes the WW domain. This proline-rich stretch functions as an intramolecular WW-interacting domain that can block the WW domain of SETD2 from interacting with the proline-rich stretch of Huntingtin, and most likely of other proteins as well.

### SRI domain

By analyzing a series of SET2-deletion-mutants, Kizer *et al* [27] identified a novel domain that specifically interacted with the hyperphosphorylated C-terminal domain (CTD) of Rpb1, the largest subunit of RNA Pol II. This Set2 Rpb1 Interacting (SRI) domain is conserved from yeast to human [27]. In human, the primary C-terminal domain-docking site of RNA Pol II is located at the first and second helices of SETD2 [9]. This domain directs the activity of SETD2 towards actively transcribed genes. Yeast experiments by Li *et al* [8] revealed a high affinity of ySET2 to the Ser2-phosphorylated CTD of RNA Pol II that is present only when transcription is well under way. ySET2 binds to the Ser5-phosphorylated CTD with intermediate affinity, while it has no affinity to the unphosphorylated CTD [28]. This interaction is dependent on the activity of the RNA Pol II CTD kinase CTK1, the enzyme responsible for the phosphorylation of Ser2 [29].

## FROM PROTEIN STRUCTURE TO BIOLOGICAL FUNCTION

**Figure 1:**
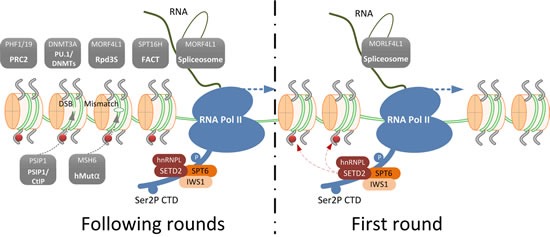
Schematic representation of SETD2-mediated trimethylation of H3K36 and an overview of the H3K36me3 readers that define its role in various biological processes During the first round of transcription, the transcription elongation factor and histone chaperone SPT6-IWS1 are recruited to Ser2P CTD tail of RNA Pol II. This results in the recruitment of the SETD2-hnRNPL complex that trimethylates H3K36. This mark is preserved on the histones in the following rounds of transcription and serves as a signal beacon to recruit H3K36me3 readers (shown in grey boxes). Facilitates Chromatin Transcription (FACT) complex, Histone deacetylase (HDAC) complex, PU.1 (also known as Spi-1 proto-oncogene, SPI1)/ DNA (cytosine-5-)-methyltransferase (DNMTs) complex and Polycomb Repressive Complex 2 (PCR2) complex are recruited for chromatin structure remodeling to facilitate transcription elongation and to prevent cryptic transcription initiation. The spliceosome is recruited through MORF4L1 for splicing selection; PSIP1/CtIP complex is recruited through PSIP1 for homologous recombination (HR) repair of double strand breaks (DSBs) and hMutα complex is recruited through MSH6 for DNA mismatch repair.

The above-mentioned functional domains define the biological function of SETD2. By virtue of its AWS-SET-PostSET domains, SETD2 mediates trimethylation of H3K36 [4]. *In vitro*, human SETD2 can carry out mono-, di-, and tri-methylation of H3K36 [30], but *in vivo* the scenario is different. While ySET2 catalyzes all methylation levels of H3K36 [7], SETD2 only modulates H3K36me3 in mammals. Knockdown of SETD2 induces a complete absence of H3K36me3 without disturbing the levels of H3K36me1 and H3K36me2 [6]. In human, trimethylation of H3K36 is carried out by a complex, of which SETD2 and Heterogeneous Nuclear Ribonucleoprotein L (hnRNPL) are the major subunits [31]. Based on these studies, it has become evident that SETD2 is solely responsible for this modification. Catalyzing H3K36 trimethylation is now regarded as the main function of SETD2. H3K36me3 is recognized by so-called readers, effector proteins that are recruited by specific histone modifications and determine the functional outcome of those modifications [32] (Table 1). A schematic representation of how SETD2-mediated-trimethylation of H3K36 is involved in various biological processes is shown in Figure 1.

**Table 1:** Overview of currently known H3K36me3 readers and their interacting domains

Gene symbol	binding domain	Function	Ref.
**BRPF1/2**	PWWP	Histone acetylation	[33, 34]
**DNMT3A/B**	PWWP	DNA methylation	[35]
**GLYR1**	PWWP	Histone methylation	[36]
**HDGF**	PWWP	DNA binding	[77]
**IWS1**	PWWP	Transcription elongation, splicing, mRNA export	[78]
**MORF4L1**	Chromo	Alternative splicing	[39, 50, 79]
**MSH6**	PWWP	DNA mismatch repair	[36, 62]
**MTF2**	Tudor	Histone methylation	[38, 53]
**MSL3**	Chromo	Histone acetylation	[80]
**MUM1**	PWWP	DNA damage repair	[34, 81]
**NSD1**	PWWP	Histone methylation	[36, 89]
**PHF1/19**	Tudor	Histone methylation	[38, 53, 82]
**PSIP1**	PWWP	Splicing and HR repair	[65, 66]
**SPT16H**	PWWP	Facilitate transcription and repress cryptic transcription	[52]
**WHSC1/L1**	PWWP	Histone methylation	[36, 51]
**ZMYND11**	PWWP	Transcription elongation	[83]

Note: BRPF1/2, Bromodomain And PHD Finger Containing 1 and 2; GLYR1, Glyoxylate Reductase 1 Homolog; HDGF, Hepatoma-Derived Growth Factor; MSH6, MutS Homolog 6; MTF2, Metal Response Element Binding Transcription Factor 2; MSL3, Male-Specific Lethal 3 Homolog; MUM1, Melanoma Associated Antigen 1;NSD1, nuclear receptor binding SET domain protein 1; PHD1/19, PHD Finger Protein 1/19; WHSC1, Wolf-Hirschhorn Syndrome Candidate 1; WHSC1L1, Wolf-Hirschhorn Syndrome Candidate 1-Like 1; ZMYND11, Zinc Finger MYND-Type Containing 11.

The most prominent function of SETD2 is thus indirectly determined by the factors that target SETD2 to specific nucleosomes to be trimethylated on the one hand, and the so-called readers of this modification on the other. Vezzoli *et al* [33] showed that BRPF1 (Bromodomain And PHD Finger Containing 1) interacts with H3K36me3 through its PWWP domain, a finding later corroborated by a study of Wu *et al* [34]. Subsequently, several other “readers” were identified that interact with H3K36me3 by virtue of their PWWP domain [35, 36, 37]. More recently, additional proteins were identified that interact with H3K36me3 through their tudor domain [38] or chromodomain [39].

In addition to its role in histone modification, SETD2 may also interact directly with other proteins, most likely through its WW domain. The BioGRID database (http://thebiogrid.org) lists multiple proteins that directly interact with SETD2. Co-immunoprecipitation assays showed that the C-terminal domain of SETD2 interacts with the N-terminal domain of TP53 [25]. Binding of SETD2 to TP53 modulates the expression of a specific set of TP53 downstream target genes, including the apoptosis related genes puma, noxa, and p53AIP1. However, because no follow-up studies have corroborated these findings, the importance of the SETD2-TP53 interaction remains to be established. Given the well-known role of TP53 in cancer development, exploring its interactions with SETD2 may be relevant to elucidating the role of SETD2 mutations in cancer. To date, most studies have focused on the SETD2-dependent trimethylation of H3K36.

### Distribution of H3K36me3

Krogan *et al* [29] were the first to report a specific distribution of H3K36me3 over the yeast genome, with enrichment of H3K36me3 in actively transcribed coding regions. In *C. elegans*, actively transcribed genes also have much higher levels of H3K36me3 than transcriptionally repressed genes [40]. The same pattern is observed in higher eukaryotes, with high H3K36me3 levels downstream of the first exon of actively transcribed genes and across the whole gene body with a peak near the 3′ end [41, 42].

In both human and mouse, intron-containing genes showed relatively higher levels of H3K36me3 than intron-less genes, irrespective of transcriptional activity [43]. Along the gene body, H3K36me3 enrichment also appears to be discrete, co-localizing to exons rather than introns, and with higher levels of H3K36me3 at constitutively included exons as compared to alternatively spliced exons [40]. The distribution pattern of H3K36me3 indicates a role for SETD2 in modulating splicing events by marking exonic and intronic regions.

It should be noted that H3K36me3 is not confined to actively transcribed genes. A study by Chantalat *et al* [44] showed a high level of H3K36me3 at the silenced Snurf-Snrpn region in mice, a well-known facultative heterochromatin domain. Pericentromeric regions, which consist mainly of constitutive heterochromatin, are also enriched for H3K36me3 [44]. In these regions the H3K36me3 mark is apparently not correlated with transcriptional events. In the remainder of this review we will discuss how the loss of or decrease in H3K36me3 caused by functional loss of SETD2 could contribute to cancer development.

## H3K36ME3-MEDIATED BIOLOGICAL FUNCTIONS

### H3K36me3 participates in transcription elongation and splicing selection

Deletion of the SRI domain of SETD2 not only abolishes its interaction with RNA Pol II but also leads to a defect in trimethylation of H3K36, suggesting that H3K36 trimethylation and transcription elongation are coupled processes [27, 28]. Splicing and transcription are also coupled processes regulated by many factors, including chromatin remodeling complexes [45], RNA Pol II elongation rate [46], RNA binding elements [47] and histone modifications [48]. Direct evidence to support participation of SETD2 in splicing came from studies on alternative splicing of the human fibroblast growth factor receptor 2 (FGFR2) gene [49]. FGFR2 is spliced into two mutually exclusive and tissue-specific isoforms: FGFR2-IIIb (exon IIIb is included) and FGFR2-IIIc (exon IIIc is included). Alternative splicing is modulated by polypyrimidine tract binding protein 1 (PTBP1, also known as PTB). PTBP1 is recruited by histone tail-binding protein Mortality Factor 4 like 1 (MORF4L1, also known as Eaf3 and MRG15), which recognizes H3K36me3 through its chromo domain [39, 50]. Overexpression of ySET2 leads to a global increase of H3K36me3 and a decreased inclusion of exon IIIb, whereas siRNA-mediated knockdown of human SETD2 resulted in inclusion of the PTB1-repressed exon IIIb [49].

This “chromatin affects splicing” model does not, however, explain by what mechanism chromatin is modified to direct splicing. Subsequently a “splicing affects chromatin” model was proposed [43]. Inhibiting splicing, either by knockdown of splicing factor Sin3A-associated protein (SAP130) or D-ribofuranosyl-benzimidazole (DRB) treatment, leads to a decreased recruitment of SETD2 and reduced H3K36me3 levels [43]. Thus, the splicing machinery itself may play a role in the recruitment of SETD2 by RNA Pol II and the subsequent trimethylation of H3K36. DRB-treatment of HeLa cells reduced the H3K36me3 levels on internal exons to a level that remained higher than the level in intergenic regions, even though both regions have a comparable RNA Pol II occupancy. This indicates that, although splicing is not required for trimethylation, it does modulate H3K36me3 levels [43]. Kim *et al* [51] showed that introducing mutations that prevent splicing, or interfere with the splicing machinery using splicing inhibitor spliceostatinA (SSA), led to a redistribution of H3K36me3 with a shift towards the 3′ region, again indicating a direct causal relationship between splicing and H3K36me3.

### H3K36me3 prevents spurious transcription

Modification of nucleosomes plays an important role in protecting genomic DNA and regulating its accessibility. A compact nucleosome structure of the gene body is needed to prevent spurious transcription initiation from cryptic promoters. Removal of this barrier during transcription elongation upon passage of RNA pol II results in a more accessible chromatin. Reconstitution of completely evicted nucleosomes with acetylated nucleosomes from the soluble pool after passage of RNA pol II could result in a more accessible chromatin structure of transcribed genes. This would allow intergenic transcription initiation from cryptic promoter sequences. Trimethylation of H3K36 during transcription elongation by RNA pol II-bound SETD2 is thought to prevent spurious transcription from cryptic promoters. H3K36me3 recruits Facilitates Chromatin Transcription complex (FACT) [52] and Polycomb repressive complex 2 (PRC2) [38, 53] to restore the repressed chromatin structure after elongation. The FACT complex disassembles the H2A-H2B dimer from the nucleosomes. After passage of RNA Pol II, the same complex promotes the replacement of the H2A-H2B dimers. This allows the transcription elongation complex to pass without the need to remove histone H4 and H3 [54]. Thus, the H3K36 trimethylated nucleosomes are kept on their position. The IWS1:SPT6:CTD complex is needed for the recruitment of SETD2 to RNA Pol II for trimethylation of H3K36 [55]. SPT6 was already known to enhance the elongation rate by displacing the nucleosomes in front of RNA pol II [56]. However, several studies have indicated that SPT6 also enhances the elongation rate in the absence of nucleosomes [57-59]. Experiments in *S. cerevisiae* have shown that inactivation of SPT6 or the FACT subunit Suppressor Of Ty 16 Homolog (SPT16H, also known as SPT16) resulted in intragenic transcription initiation events from cryptic promoters [52, 60, 61]. Taken together, the prevention of spurious intragenic transcription initiation is an important function of H3K36me3 and thus, indirectly, of SETD2.

### H3K36me3 maintains genomic integrity and stability

The enriched level of H3K36me3 in transcribed regions not only serves to restore chromatin structure after transcription but also functions in maintaining genomic integrity. H3K36me3 is a crucial factor in the repair of DNA damage in transcribed regions by modulating two different pathways: (i) the DNA Mismatch Repair (MMR) pathway responsible for the repair of nucleotide mismatches and small insertion/deletion loops of simple repeat sequences and (ii) the homologous recombination (HR) repair of DNA double strand breaks (DSBs).

DNA MMR is a mechanism for correcting base-base mismatches and insertion/deletion loops produced during replication. The most abundant machinery responsible for DNA MMR is the hMutSα (MSH2-MSH6) complex. Li *et al* showed that the binding of hMutSα to chromatin is H3K36me3-dependent as its subunit MSH6 reads the H3K36me3 signal by virtue of its PWWP domain [62]. Depletion of SETD2 abolished the localization of hMutSα, which led to a DNA-MMR-deficient mutator phenotype. The DNA MMR defect in SETD2-deficient UOK143 cells could be restored by enforced expression of ySET2. This demonstrates the crucial role of H3K36me3 in recruiting the DNA MMR repairing machinery.

DNA MMR predominantly occurs during the S-phase of the cell cycle, whereas HR repair preferentially takes place in the late S/G2 phase. H3K36me3 consistently peaks in the late G1/early S phase and disappears in the late S/G2 phase [63] (reviewed by Li *et al* [64]). This is additional proof that H3K36me3-modification enables a safe transition from the G1 to the S phase by recruiting repairing machineries to correct the errors produced during replication. When H3K36me3 is abolished due to SETD2 inactivation, the repair machinery cannot localize to damaged sites, resulting in an accumulation of errors and genomic instability, a hallmark of tumorigenesis.

H3K36me3 also serves as a signal to recruit proteins to DNA double strand breaks (DSBs) to initiate repair. An accurate repair of DSBs relies on HR. The PWWP domain of PC4 And SFRS1 Interacting Protein 1 (PSIP1, also known as Lens Epithelium-Derived Growth Factor, LEDGF) is the basis of this HR process, and H3K36me3 plays a key role through the recruitment of PSIP1 [65-67]. This is consistent with the finding that SETD2 is required for ATM-activation upon DSBs [68] and the notion that SETD2-deficient cells fail to activate a proper DNA damage response, including activation of TP53 [68]. SETD2 inactivation abolishes H3K36me3 and consequently the binding of PSIP1 to the damage sites. To compensate for the HR deficiency, cells have to use alternative mechanisms to repair the DSBs, such as nonhomologous end-joining and/or microhomology-mediated end-joining [68]. These approaches are error prone and may lead to deletions [69]. Although the HR repair machinery in SETD2-inactivated cells is still competent [67, 68], these cells are not capable of recruiting the DNA repair components to the damaged sites due to loss of the H3K36me3 signal.

### H3K36me3 and DNA methylation

Several publications have indicated that actively transcribed genes are extensively methylated at the gene body [70-73]. This has raised the question of whether H3K36 trimethylation is associated with gene body DNA methylation. Hahn *et al* [74] carried out a detailed study of the association of a number of epigenetic markers in human bronchial epithelial cells and colorectal cancer cell line HCT116, focusing on chromosome 19 genes. Of the expressed genes, 74% had a high level of both gene body DNA methylation and H3K36me3. DNA methylation and H3K36me3 have been linked in both yeast and mouse [75]. In addition, a group of genes, mostly Zinc Finger genes, were identified in which H3K36me3 occurred in combination with the repressive intragenic H3K9me3 mark [75]. On average these genes were expressed at a low level and had a relatively low number of intragenic CpG dinucleotides that were largely unmethylated. By analyzing cells that were either made defective in H3K36 trimethylation or in CpG methylation, Hahn *et al* [74] further showed that the levels of these two epigenetic markers are established independently. However, Dhalayan *et al* demonstrated a high affinity of DNA (cytosine-5)-methyltransferase 3A (DNMT3A) to H3K36me3 *in vitro* [35]. DNMT3A is targeted to H3K36me3-containing nucleosomes, e.g. in heterochromatic regions as well as gene bodies, by virtue of its PWWP domain. DNMT3A/B interacts with PU.1 to form a complex for *de novo* site-specific methylation [76]. This indicates that the H3K36me3 mark could recruit DNMT3A/B to establish DNA methylation.

### SETD2 knock-out mouse

In mice, *SETD2*−/− knockout is embryonic lethal in E10.5 to E11.5 due to defects in angiogenesis in the yolk sac and placenta [84]. Expression profiling of *SETD2*−/− and wild-type yolk sacs revealed significantly altered expression levels of genes involved in vascular remodeling. Both *SETD2*−/− embryonic bodies derived from embryonic stem cells and from cultured human endothelial cells treated with siRNAs-directed against *SETD2* showed defects in cell migration and invasion [84, 85]. Thus, SETD2 appears to be crucial for a proper embryonic development although many cancer cells appear to function well without SETD2. In the literature, no clues can be found of heterozygous *SETD2* knockout mice being predisposed to any kind of disease or cancer.

## SETD2 IN DISEASE

Luscan *et al* [86] identified a missense and a nonsense *SETD2* mutation in 2 out of 11 patients with Sotos syndrome, an overgrowth syndrome first described by Sotos *et al* [87]. It is unknown if these mutations were present in the germline and there is no direct functional evidence that links these mutations to SOTOS. However, it is remarkable that the gene most frequently mutated in SOTOS is the PWWP-domain-containing Nuclear Receptor Binding SET Domain Protein 1 (NSD1, also known as KMT3B) gene [88] responsible for mono- and di-methylation of H3K36 [30, 89]. We are not aware of any reports that link SETD2 germline mutations to an inherited syndrome in humans.

## SETD2 IN CANCER

The first report on *SETD2* mutations in cancer dates from 2010 when Dalgliesh *et al* identified inactivation mutations in ccRCC [90]. At the same time, using a “Gene Identification by Nonsense-mediated mRNA decay Inhibition (GINI)” strategy, our group identified inactivating *SETD2* mutations in 5 out of 10 ccRCC-derived cell lines [91]. All cell lines showed copy number loss for most of the short arm of chromosome 3, indicating complete functional loss of SETD2 in these cell lines. Subsequent targeted sequencing of the *SETD2* coding regions revealed *SETD2* mutations in 2 out of 10 primary ccRCC tumors [92]. This bi-allelic inactivation of SETD2 was the first clue that the gene might be a tumor suppressor gene. Two large cohort studies revealed an overall frequency of *SETD2* mutations of approximately 11% in ccRCC [93, 94]. The fraction of truncating mutations in ccRCC was more than 50% in the study of Hakimi *et al* [95] and 57% in COSMIC, which is significantly higher than the fraction of truncating mutations in non-ccRCC tumors (32%, COSMIC). Still, whole-exome sequencing studies did reveal somatic *SETD2* mutations in various types of cancer (Table 2), and this can be seen as an indication that SETD2 inactivation plays a role in the development of other tumors, albeit with low frequencies in most of them (COSMIC [96], Tumorportal [97] and cBIOPortal [98, 99], accessed in January 2016). It should be noted that in many studies it is not clear if the mutation resulted in a bi-allelic inactivation of *SETD2*. Moreover, the majority of somatic *SETD2* mutations were missense mutations for which the functional consequences are often unclear (Table 2). This is illustrated by the study of Zhu *et al* [100] of 241 cases of leukemia (134x acute myeloid leukemia (AML) and 107x acute lymphcytic leukemia (ALL)) in which only 8 of the 19 somatic SETD2 mutations identified in 15 patients were truncating. Bi-allelic mutations were detected in only 4 patients. It cannot be excluded that in ALL, and possibly in other tumors as well, *SETD2* haploinsufficiency does lead to a disease phenotype. *SETD2* mutations appeared to be most frequent in leukemias that carried a *MLL* gene rearrangement [100].

**Table 2:** Overview of SETD2 mutation frequencies in a selection of tumors based on the COSMIC database (Feb 2016)*

Tissue/tumour subtype	Percentage of samples with mutation		cases tested
	truncating	missense	
**Kidney**	**4.19**	**3.10**	**2197**
*ccRCC*	*5.43*	*4.14*	*1473*
**Lung**	**1.26**	**1.42**	**1826**
*Adenocarcinoma*	*3.51*	*3.51*	*550*
**Skin**	**1.08**	**2.65**	**1017**
**Liver**	**0.74**	**1.55**	**1611**
*Hepatocellular* carcinoma	*0.78*	*1.12*	*893*
**Soft tissue**	**0.70**	**4.67**	**428**
**Biliary tract**	**0.66**	**0.66**	**152**
*Adenocarcinoma*	*0.67*	*0.67*	*150*
**Endometrium**	**0.63**	**3.49**	**631**
*Endometrioid carcinoma*	*0.74*	*4.08*	*539*
**Large intestine**	**0.59**	**3.05**	**1345**
*Adenocarcinoma*	*0.62*	*3.10*	*1298*
**Breast**	**0.58**	**0.94**	**1378**
**Central nervous system**	**0.47**	**0.38**	**2128**
**Pancreas**	**0.46**	**0.33**	**1521**
*Ductal carcinoma*	*0.40*	*0.57*	*1240*
**Stomach**	**0.34**	**2.04**	**587**
**Urinary tract**	**0.30**	**0.90**	**666**
**Haematopoietic and lymphoid**	**0.24**	**0.87**	**2519**
*Acute lymphoblastic B cell leukaemia*	*1.54*	*2.32*	*258*
*Acute lymphoblastic T cell leukaemia*	*0.97*	*0.97*	*207*
*Diffuse large B cell lymphoma*	*0.00*	*3.20*	*250*
**Ovary**	**0.24**	**0.59**	**843**
*Serous carcinoma*	*0.31*	*0.78*	*641*
**Bone**	**0.20**	**0.60**	**496**
**Prostate**	**0.10**	**0.88**	**1019**
*Adenocarcinoma*	*0.12*	*0.48*	*827*

* Tumor subtypes with a sample size less than 100 cases have been excluded.

**Figure 2:**
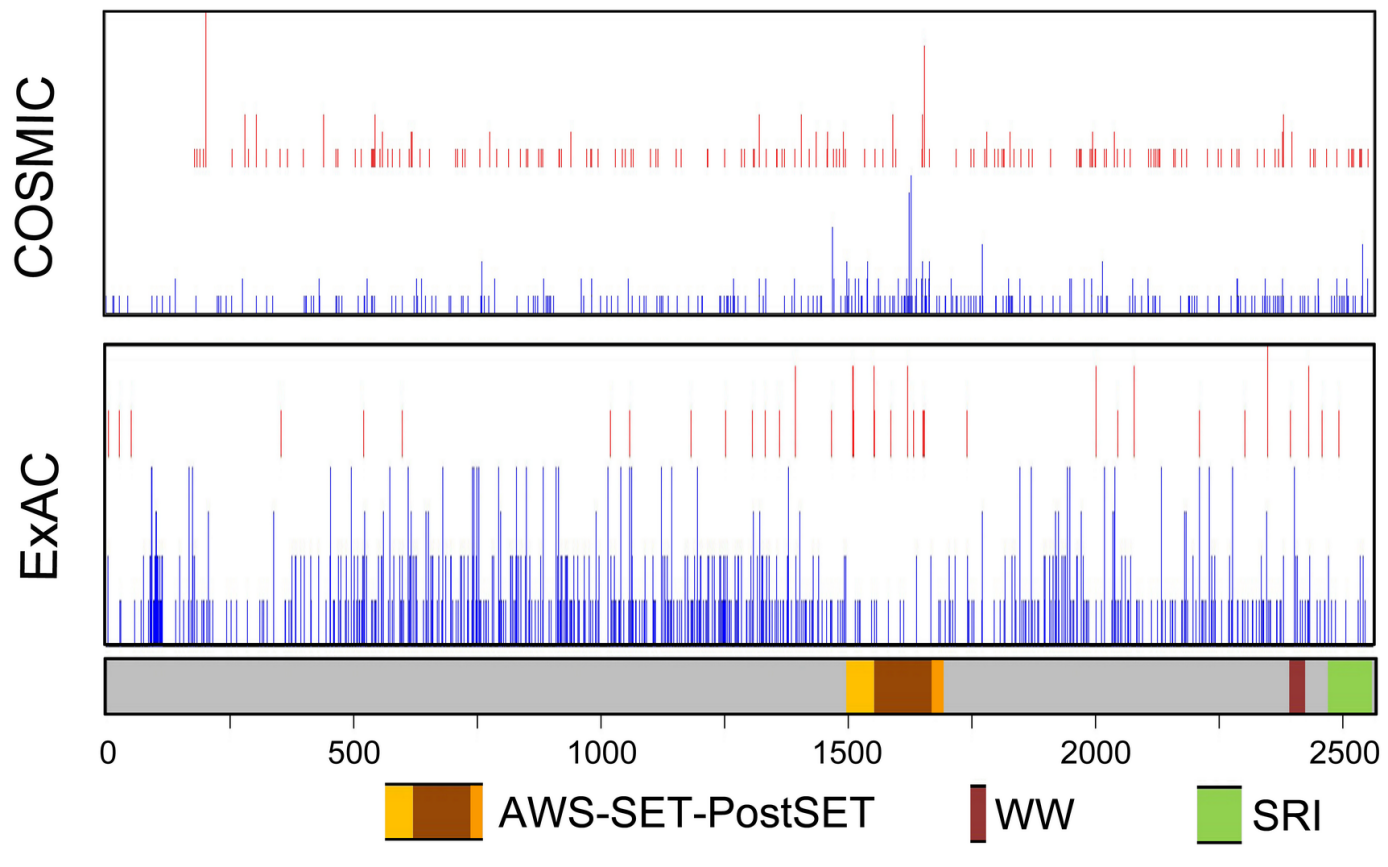
Schematic representation of SETD2 with the location of functional domains and nonsynonymous mutations and variants The location of nonsynonymous mutations was obtained from ExAC (Germline variants in ~120000 alleles; January 2016) and COSMIC (somatic variants in 23,249 cases; January 2016). Intronic regions and 3′- and 5′-untranslated regions are not shown. Red, position of inactivating variants; Blue, position of missense variants. For the COSMIC data, the height of the bar is relative to the number of mutations. For the ExAC data, the height of the bars indicate 1, 2-5, 6-10, or >10 variants per triplet.

In ccRCC, *SETD2* is ranked in the top-5 most commonly mutated genes (COSMIC, rank 4), indicating its specific role in this tumor type. In Tumorportal, *SETD2* mutations are indicated as “highly significant” in ccRCC and glioblastoma multiform and indicated as “near significant” in bladder cancer. In all cancers combined, there is a slight clustering of *SETD2* missense mutations in an approximately 200 amino acid segment (p.M1468 up to p.Q1668) that overlaps with the SET domain (Figure 2). The same region is relatively devoid of missense variants in the normal population (ExAC database, http://exac.broadinstitute.org, accessed January 2016, and Figure 2), indicating that missense mutations in this domain might be more often damaging. *SETD2* nonsense mutations leading to loss-of-function can be located throughout the entire gene (Figure 2). Further studies on the potential functional consequences of *SETD2* missense mutations are required to establish their role in tumor development and/or progression.

Pena-Llopis *et al* [101] collected data on 924 primary ccRCC of which 300 cases had a PBRM1 mutation and 66 cases had a *SETD2* mutation, while 33 cases had a mutation in both genes. This number was shown to be significantly higher than the expected number of cases with mutations in both genes (*n* = 21, Fisher exact test, *p* = 0.003). This suggests that mutations of PBMR1 and SETD2 may have a synergistic effect in ccRCC, possibly by disrupting different pathways. Moreover, the Cancer Genome Atlas database (TCGA) reveals co-mutation of *PBRM1* and *SETD2* in multiple tumors despite the low mutation frequency of both genes in these cancers. Thus, having both *SETD2* and *PBRM1* mutations might strengthen their oncogenic potential, and the underlying mechanism deserves exploration. Sato *et al* [94] found that *SETD2* mutations predominantly occurred in tumors with pre-existing VHL mutations, again indicating a role in tumor progression. However, in other studies *SETD2* mutations were also identified in ccRCC cases with wild type VHL [95, 102].

The high frequency of inactivating *SETD2* mutations in ccRCC points to a tumor-suppressor-like function of this gene. Additional proof for a tumor suppressor role of SETD2 came from Sleeping Beauty transposon experiments. This approach is based on the assumption that commonly observed transposon insertion sites can harbor tumor-driver genes. These studies revealed transposon integration sites in *SETD2* in various tumors such as leukemia's [103] and colorectal cancer [104], albeit at a low frequency.

### Correlation with clinical data

Al Sarakbi *et al* [105] found a negative association of *SETD2* expression levels with increasing tumor stage in breast cancers. In gliomas, *SETD2* mutations were predominantly seen in high-grade (16 out of 178 cases) but not in low-grade cases (0 out of 45 cases) [106]. ccRCC patients with somatic *SETD2* mutations had a higher relapse rate compared to cases with wild-type *SETD2*, but no effect was observed on overall survival. In a study including 185 ccRCC patients, *SETD2* mutations were significantly associated with advanced tumor stage (*P* = 0.02) [95]. In the TCGA, *SETD2* mutations were found to be associated with worse cancer-specific survival (*P* = 0.036; HR 1.68; 95% CI 1.04-2.73), and the presence of *SETD2* mutations was a predictor of ccRCC recurrence in an univariant analysis (*P* = 0.002; HR 2.5; 95% CI 1.38-4.5) [107]. Further evidence supporting a role of SETD2 inactivation in progression of tumors comes from a recent study performed by Ho *et al* [108]. Using immuno-histochemical approaches, Ho *et al* (108) observed a decrease in H3K36me3 levels in metastatic ccRCC as compared to primary ccRCC. Either acquired *SETD2* mutations or alternate mechanisms may be the cause of this, suggesting that a decreased level of H3K36me3 is correlated with progression. They also noted that loss of one allele of *SETD2*, a common event due to the widespread copy number loss of the short arm of chromosome 3 in ccRCC, did not result in a reduced level of H3K36me3. Thus, *SETD2* haploinsufficiency does not cause a H3K36me3-related phenotype in ccRCC. In addition, intra-tumor heterogeneity studies have indicated that SETD2-inactivation may be a late event in cancer development. Gerlinger *et al* [109] carried out a genomic analysis of multiple regions of four primary ccRCC tumors and detected intratumor heterogeneity in every case. Using whole exome sequencing and H3K36me3-staining of tissue sections, they identified different *SETD2* mutations in different regions of the same primary tumor in three cases. This suggested that loss of SETD2 can be a late event that provides a selective advantage to tumor cells [109]. Lentiviral-mediated knockdown of *SETD2* in pre-leukemic cells carrying a MLL fusion-gene increased both the colony-forming capacity and the growth rate of these cells [100]. This indicates that loss of functional SETD2 facilitates initiation as well as progression of leukemias. Thus, it appears that SETD2-inactivation may function not only in driving the development of tumors, but also in promoting progression of the disease.

### SETD2 functional studies in cancer

Alternative splicing is considered as a major impetus driving proteome diversity and promoting progression of cancer [110]. *SETD2*-mutated ccRCC tumors showed an altered chromatin accessibility in the H3K36me3 marked regions, which led to widespread defects in transcript processing, including intron retention, exon utilization and different transcriptional start and stop site usage, especially in highly expressed genes [111]. A specific set of transcripts showed an increased retention of introns in H3K36me3-deficient tumors, and several of the affected genes, including PTEN, TP53, ATR, RAD50, POLN, XRCC1, CCNB1, and CCND3, are important in tumor development. Since intron retention could lead to loss of function of the protein product, SETD2-inactivation will probably also have an impact on the functionality of these genes. Additionally, in the study of Ho *et al* [108], decreased levels of H3K36me3 in ccRCC, most likely due to *SETD2*-inactivating mutations, resulted in alternative exon usage for a selection of genes [108]. Li *et al* [112] carried out a detailed study on the splicing of CDH1 in gastric cancer cell lines in comparison to the human gastric mucosal epithelial cell line GES-1. In all samples, the wild type product and a transcript that lacks part of exon 8 were identified. A higher level of H3K36me3 appeared to favor the use of the splice donor site within exon 8. Attempts to influence the ratio between the two transcript variants were most successful using siRNA directed against SETD2 and, to a lesser extent, using an HDAC inhibitor.

HR repair and DNA MMR defects have been observed in SETD2-inactivated tumor cell lines, although the repair machineries themselves are not abolished in these cells [62, 67]. The SETD2-deficient ccRCC-derived cell line UOK143 showed insufficient MutSα-mediated DNA MMR in S phase. In contrast, in the SETD2-proficient ccRCC cell line UOK12, abundant MSH6 foci were formed during S phase and most of those loci co-localized with the H3K36me3 signal. SETD2-inactivated ccRCC cell lines RCC-MF and RCC-FG2 showed defects in DSB repair [68]. These studies indicated that SETD2 is important to maintain the genomic integrity in ccRCC.

### Additional factors modulating H3K36me3 levels

**Figure 3:**
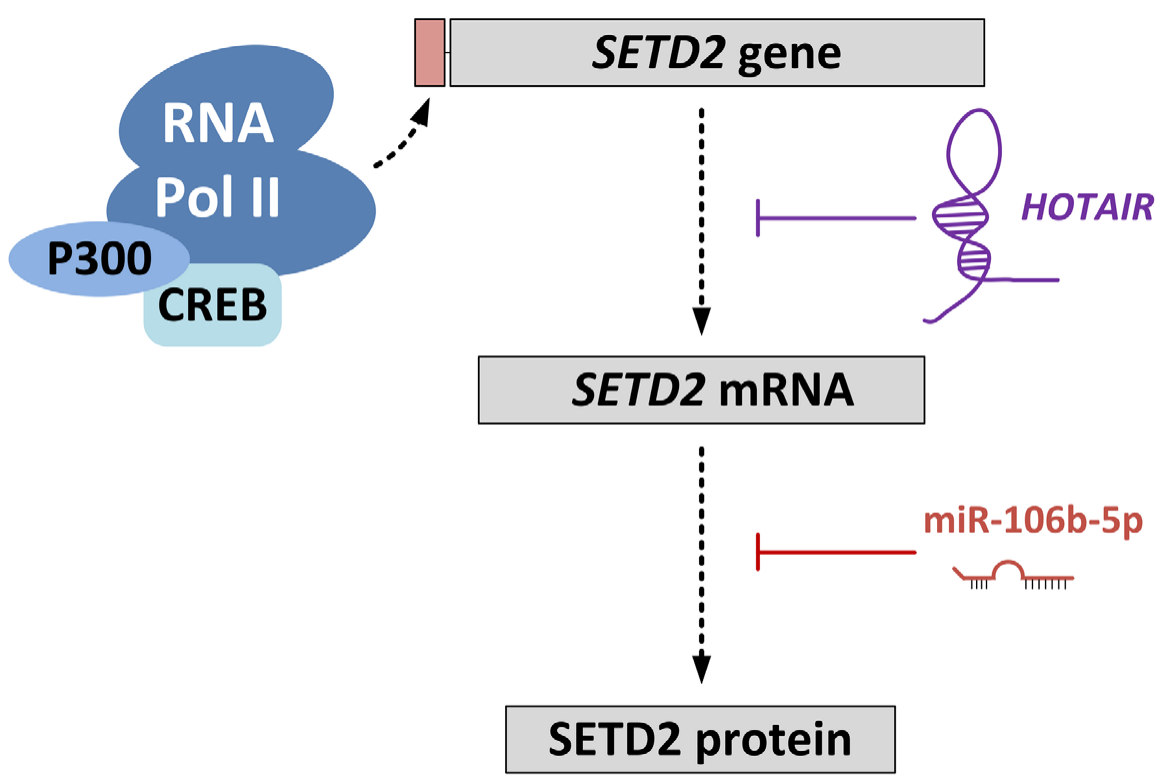
Regulation of SETD2 expression The long non-coding RNA HOTAIR regulates *SETD2* expression at the transcriptional level by competitively blocking loading of CREB-P300-RNA Pol II complex to the *SETD2* promoter. MicroRNA-106-5p (miR-106-5p) regulates *SETD2* expression at the translational level by binding to the 3′-UTR of the *SETD2* mRNA transcript.

When examining several databases, it becomes clear that SETD2 is ubiquitously expressed in most if not all tissues. This is not surprising given its function as the sole gene responsible for the trimethylation of H3K36. However, two factors have been identified in cancer-related studies that can modulate the level of SETD2 in cancer cells, and may also do so in non-cancerous cells (Figure 3). A recent study on liver cancer demonstrated a negative correlation between expression of SETD2 and the HOX transcript antisense RNA (HOTAIR) [116]. HOTAIR expression has been associated with several cancers and is shown to be an oncogenic long noncoding RNA [117]. HOTAIR suppressed the transcription of SETD2, and reduced the level of H3K36me3. Thus, HOTAIR overexpression is linked to various cellular processes mediated by H3K36me3 readers.

Xiang et al [118] showed that miR-106b-5p could bind to, and inhibit translation of, the SETD2 mRNA transcript in ccRCC. SETD2 levels increased by inhibiting miR-106b-5p and this resulted in suppression of cell proliferation and a G0/G1 cell cycle arrest.

A number of genes other than SETD2 can influence H3K36me3 levels. KDM4A, -B and -C are known to demethylate H3k36me3 [113]. Overexpression of these genes, which is a relative common event in various types of cancer [114], may thus interfere with all processes that involve H3K36me3 readers. As an example, it was recently shown that an enhanced expression of KDM4A-C promotes genomic instability [115]. By demethylating H3K36me3 the recruitment of MSH6 is prevented.

## EMERGING THERAPEUTIC OPPORTUNITIES

Now that it is evident that SETD2-inactivation can be an important factor in tumor development and progression, especially in ccRCC, understanding the SETD2-inactivation-related pathways may offer new targets for therapy. The Genomics of Drug Sensitivity in Cancer database [119] lists four chemical compounds with a selective inhibitory capacity for SETD2−/− cell lines. Two of these components target P13Kbeta. Feng *et al* [120] further analyzed the effects of AZD6482 on SETD2−/− ccRCC cell lines and showed that tumor cells were selectively inhibited. This represents the first indication that novel compounds targeting SETD2−/− tumors might become feasible treatment for ccRCC patients. In recent years many studies have focused on the ability of small molecules to target specific histone modifications, which could eventually be used in targeted therapies. A recent study shows that the combination of WEE1-inactivation by the AZD1775 inhibitor and H3K36me3-deficiency is lethal for cultured human cells [121]. These results were then validated in xenograft models of two tumor-derived SETD2−/− cell lines. The underlying mechanism appears to be inhibition of the replication process. These recent developments may open the doors that allow for the development of targeted therapies for H3K36me3-deficient tumors in combination with WEE1 inhibitors. The WEE1 inhibitor is currently being tested in several phase II clinical trials (http://www.clinicaltrials.gov).

## CONCLUDING REMARKS

SETD2 is responsible for the trimethylation of H3K36 in the gene body of actively transcribed genes and its inactivation interferes with the function of readers of this specific histone modification. The role of H3K36me3 on specific cellular functions is becoming more and more clear. Loss of one allele of *SETD2*, most likely a common event in many tumors due to widespread and frequent 3p copy number loss, may not be enough to cause a significant change in H3K36me3. On the other hand, biallelic inactivation of SETD2 is not the only mechanism that may cause loss of H3K36me3. Loss of SETD2 may also cause regional genomic instability, RNA processing defects and intragenic transcription initiations. Both genomic instability and alternative splicing are known as hallmarks of cancer. The former is a key force in carcinogenesis. The latter is an important mechanism for driving proteome diversity, which contributes to cancer development. In combination with the presence of *SETD2*-inactivating mutations in a substantial proportion of ccRCC, this clearly demonstrates SETD2′s role as a suppressor of both tumor initiation and progression.

Our knowledge on SETD2-regulated signaling pathways is quite limited, especially in the context of SETD2 binding proteins. Recent studies have indicated that SETD2 may interact with multiple proteins [122-124]. The challenge will be to unravel novel SETD2 functionalities that are independent of its function as trimethylator of H3K36. Conditional, and/or tissue-specific, SETD2 knockout mice may be of help to identify the crucial pathways that are affected upon inactivation of SETD2. Loss of SETD2 appears to play an essential role in a substantial subset of ccRCC. However, the specific effect of SETD2 inactivation on ccRCC precursor cells, kidney primary tubular epithelial cells, is still unknown. As *SETD2* mutations are also seen in other cancer types, understanding the role of SETD2 in ccRCC will contribute to our understanding of these tumors.
